# Colorectal Cancer After Screening Colonoscopy: 10-Year Incidence by Site and Detection Rate at First Repeat Colonoscopy

**DOI:** 10.14309/ctg.0000000000000535

**Published:** 2022-10-06

**Authors:** Sarina Schwarz, Michel Hornschuch, Christian Pox, Ulrike Haug

**Affiliations:** 1 Department of Clinical Epidemiology, Leibniz Institute for Prevention Research and Epidemiology, BIPS, Bremen, Germany;; 2 Department of Medicine St. Joseph-Stift Bremen, Bremen, Germany;; 3 Faculty of Human and Health Sciences, University of Bremen, Bremen, Germany.

## Abstract

We aimed to describe cumulative colorectal cancer (CRC) incidence after screening colonoscopy stratified by tumor location, age, and sex as well as CRC detection rate at first repeat colonoscopy.

**METHODS:**

Using the German Pharmacoepidemiological Research Database, we included persons with screening colonoscopy and assessed cumulative CRC incidence after baseline screening colonoscopy with snare polypectomy (cohort 1) and without polypectomy (cohort 2). We also determined the CRC detection rate at first repeat colonoscopy by time since screening colonoscopy.

**RESULTS:**

Overall, 1,095,381 persons were included. The 10-year cumulative CRC incidence was 1.5% in cohort 1 and 0.6% in cohort 2. The proportion of proximal CRC increased with age: In women of cohort 1, 47% of CRCs in the age group 55–64 years were proximal (men: 42%) while in the age group 65–74 years, this proportion was 55% (men: 49%). In cohort 2, similar patterns were observed. In cohort 1, the CRC detection rate at first repeat colonoscopy among persons examined within 6–8 years after screening colonoscopy was more than twice as high compared with those examined within 4–6 years (1.7% vs 0.8%).

**DISCUSSION:**

Among persons followed up after screening colonoscopy, we observed a steadily increasing predominance of proximal CRC, and this shift showed distinct patterns by age and sex. Because our study suggests higher CRC detection rates among persons with a later repeat colonoscopy, the role of delayed surveillance and the benefit of a reminder system should be explored.

## INTRODUCTION

Colonoscopy has been shown to reduce colorectal cancer (CRC) incidence and mortality (1). Nonetheless, colonoscopy is not perfect, and CRC also occurs among persons with prior colonoscopy. Several cohort studies have described proximal and distal CRC incidence after colonoscopy (2–10). For example, Nishihara et al. (4) including approximately 40,000 persons undergoing sigmoidoscopy or colonoscopy and followed over 22 years reported a higher incidence of proximal as compared with distal CRC in persons with prior polypectomy (40% higher), negative sigmoidoscopy (30% higher), and negative colonoscopy (100% higher). However, neither the sample size of the study by Nishihara nor of other studies on this topic allowed for further stratification, e.g., by age and sex, although it is known that the occurrence of distal and proximal CRCs generally varies according to these factors (11–14). Furthermore, existing studies reporting on cumulative CRC incidence by time interval since colonoscopy typically did not have information on CRC detection rates at first repeat colonoscopy. A description of CRC detection rate according to the time interval since screening colonoscopy may provide valuable additional insights into the temporal patterns of CRC occurrence after colonoscopy.

German claims data may partly fill these gaps. Although these data are limited regarding information on findings at colonoscopy—apart from CRC diagnoses, there is only information on whether polypectomy was performed (15)—they offer a large sample size and a long follow-up. In addition, they include information on utilization of repeat colonoscopy with an exact date of the respective examination. We, therefore, aimed to use the potential of German claims data to describe 10-year cumulative CRC incidence among persons undergoing screening colonoscopy stratified by age, sex, and tumor location as well as CRC detection rates at first repeat colonoscopy according to time since screening colonoscopy.

## METHODS

### Data source

We used the German Pharmacoepidemiological Research Database (GePaRD), which is based on claims data from 4 statutory health insurance providers in Germany and currently includes information on approximately 25 million persons who have been insured with one of the participating providers since 2004 or later. In addition to demographic data, GePaRD contains information on outpatient and inpatient services and diagnoses and on drug dispensations. Per data year, there is information on approximately 20% of the general population, and all geographical regions of Germany are represented.

The German CRC screening program is based on fecal occult blood testing (offered annually from age 50–54 years and biennially from 55 years onward) and screening colonoscopy (offered from 55 years onward; since 2019, it has been offered to men from 50 years onward). Screening colonoscopy can be distinguished from diagnostic colonoscopy because there are different reimbursement codes for these procedures. GePaRD does not contain information on number, size, and histology of polyps removed during a colonoscopy, but based on procedure and diagnosis codes, we have developed an algorithm to distinguish between persons with snare polypectomy (reimbursable for removing lesions >5 mm in size), persons with forceps polypectomy, and persons without polypectomy. Comparison of this classification with the distribution of snare polypectomy and forceps polypectomy observed in the German colonoscopy registry showed very good agreement (15). CRC diagnoses in GePaRD are coded according to the German modification of the *International Classification of Diseases, 10th revision* (*ICD-10-GM*). We considered inpatient diagnosis codes of CRC, which are considered to have a high validity. Patients with only outpatient diagnosis codes of CRC were only classified as CRC cases if additional criteria such as coding of diagnostic or surveillance examinations were fulfilled to avoid misclassification. We based this algorithm on the guidelines for handling low-risk pT1 CRCs, recommending frequent surveillance endoscopy within 6 months and within 2 years (16). Regarding the classification of CRC location into proximal and distal to the splenic flexure, we used the information as provided by the *ICD* code. CRCs with unclear information on location (C18.8 and C18.9) or persons with 2 or more CRC codes providing discordant information regarding proximal vs distal location were classified into the category “both/unknown.” Stage at diagnosis was roughly estimated based on *ICD* codes indicating lymph node involvement or distant metastases as previously described (17). In addition, we considered codes for cancer treatment typically used in more advanced stages. Based on this information, CRCs were classified into the categories “advanced” and “nonadvanced.” As presented in Supplementary Digital Content 1 (see Supplement, http://links.lww.com/CTG/A883), CRC incidence, the proportion of advanced stages, and the distribution by location in GePaRD agreed very well with cancer registry data. All codes used in this analysis are available on request.

### Study population and study design

We included all persons aged 55 years and older who underwent a screening colonoscopy between 2006 and 2017. We excluded persons who had not been continuously insured for at least 2 years before undergoing screening colonoscopy, persons without valid information on age or sex, and persons with residency outside Germany. We also excluded all persons with codes indicating prevalent CRC any time before screening colonoscopy, taking also into account status post diagnosis codes and codes indicating follow-up care in CRC survivors. We described findings at baseline screening colonoscopy for all included persons. Follow-up focused on persons with a snare polypectomy (cohort 1) and persons without polypectomy (cohort 2), i.e., 2 groups that were expected to clearly differ regarding future CRC risk. We followed each person until CRC diagnosis, end of the study period, end of insurance, or death, whichever occurred first. CRC diagnoses coded within 6 months after a colonoscopy were considered as having been detected by this colonoscopy (18). We assessed the cumulative CRC incidence during follow-up and the CRC detection rate at first repeat colonoscopy. To determine CRC detection rates at first repeat colonoscopy, we included persons with at least 1 repeat colonoscopy within more than 6 months after the baseline screening colonoscopy and at least 6 months of follow-up after the repeat colonoscopy (except if they died).

### Data analysis

In the first step, we described the findings at baseline screening colonoscopy stratified by age and sex. As described above, follow-up then focused on persons with a snare polypectomy (cohort 1) and without polypectomy (cohort 2). We described both cohorts regarding age at baseline, sex, months of follow-up and utilization of repeat colonoscopy. We then determined the cumulative incidence of total, distal, and proximal CRCs in both cohorts stratified by sex and age using Kaplan-Meier analysis. In sensitivity analyses, distal CRCs were further subdivided into cancers in the distal colon vs rectal cancer.

Furthermore, we determined CRC detection rates at first repeat colonoscopy according to time since screening colonoscopy. For example, for the first time window, all persons with a first repeat colonoscopy between 0.5 and 2 years after screening colonoscopy were in the denominator and, of these, persons with an incident CRC diagnosis detected at this repeat colonoscopy were in the numerator. Similarly, this was calculated for each 2-year interval after year 2. We also determined the stage distribution of CRCs detected at first repeat colonoscopy (advanced vs nonadvanced). We calculated 95% confidence intervals for proportions assuming binomial distributions and 95% confidence intervals using the substitution method for incidence rates (19).

## RESULTS

Overall, we included 1,095,381 persons with a screening colonoscopy between 2006 and 2017 (see Supplementary Digital Content 2, http://links.lww.com/CTG/A883). Table 1 summarizes the findings at baseline screening colonoscopy (including diagnoses coded within 6 months after colonoscopy, see methods section). Overall, 7,052 men (1.4%) and 4,794 women (0.8%) were diagnosed with a CRC at baseline, and 26% of these CRCs were in the proximal colon. The proportion of proximal CRCs was higher in older age groups, and it was higher in women than in men. Among women, it was 27% in the age group 55–64 years (men: 17%), 33% in the age group 65–74 years (men: 23%), and 37% in the age group 75+ years (men: 27%). Across all age and sex groups, approximately 30% of CRCs diagnosed at baseline were classified as advanced. Regarding other findings at baseline, 18% of all persons (N = 193,745) had a snare polypectomy and were thus assigned to cohort 1; 66% of all persons (N = 723,880) had no polypectomy and were thus assigned to cohort 2. The remaining 165,910 persons were classified as having a forceps polypectomy at baseline.

**Table 1.** Baseline findings/procedures in persons with screening colonoscopy between 2006 and 2017 by age at screening colonoscopy

	No. of persons	No polyps (row %)	Polyps		CRC					
			Snare polypectomy (row %)	Forceps polypectomy (row %)	Total CRC (row %)	Site			Stage	
						Proximal (% of total CRC)	Distal (% of total CRC)	Both/unknown (% of total CRC)	Advanced (% of total CRC)	Nonadvanced (% of total CRC)
All persons	1,095,381	723,880 (66.1%)	193,745 (17.7%)	165,910 (15.1%)	11,846 (1.1%)	3,090 (26.1%)	8,107 (68.4%)	649 (5.5%)	3,532 (29.8%)	8,314 (70.2%)
Men										
All age groups	505,934	302,862 (59.9%)	110,658 (21.9%)	85,362 (16.9%)	7,052 (1.4%)	1,557 (22.1%)	5,109 (72.4%)	386 (5.5%)	2,140 (30.3%)	4,912 (69.7%)
55 to <65 yr	273,711	168,905 (61.7%)	55,754 (20.4%)	46,814 (17.1%)	2,238 (0.8%)	389 (17.4%)	1,715 (76.6%)	134 (6.0%)	684 (30.6%)	1,554 (69.4%)
65 to <75 yr	179,368	103,612 (57.8%)	42,431 (23.7%)	30,111 (16.8%)	3,214 (1.8%)	742 (23.1%)	2,310 (71.9%)	162 (5.0%)	960 (29.9%)	2,254 (70.1%)
75+ yr	52,855	30,345 (57.4%)	12,473 (23.6%)	8,437 (16.0%)	1,600 (3.0%)	426 (26.6%)	1,084 (67.8%)	90 (5.6%)	496 (31.0%)	1,104 (69.0%)
Women										
All age groups	589,447	421,018 (71.4%)	83,087 (14.1%)	80,548 (13.7%)	4,794 (0.8%)	1,533 (32.0%)	2,998 (62.5%)	263 (5.5%)	1,392 (29.0%)	3,402 (71.0%)
55 to <65 yr	337,468	246,850 (73.1%)	43,623 (12.9%)	45,432 (13.5%)	1,563 (0.5%)	417 (26.7%)	1,051 (67.2%)	95 (6.1%)	458 (29.3%)	1,105 (70.7%)
65 to <75 yr	197,448	137,429 (69.6%)	30,147 (15.3%)	27,809 (14.1%)	2,063 (1.0%)	683 (33.1%)	1,263 (61.2%)	117 (5.7%)	603 (29.2%)	1,460 (70.8%)
75+ yr	54,531	36,739 (67.4%)	9,317 (17.1%)	7,307 (13.4%)	1,168 (2.1%)	433 (37.1%)	684 (58.6%)	51 (4.4%)	331 (28.3%)	837 (71.7%)

CRC, colorectal cancer.

Table 2 characterizes persons in cohorts 1 and 2 regarding age, sex, length of follow-up, and utilization of repeat colonoscopy. The mean age was 65 years in cohort 1 and 64 years in cohort 2. The proportion of women was lower in cohort 1 (43%) than in cohort 2 (58%). Approximately half of the persons in cohort 1 (47%) had at least one repeat colonoscopy during follow-up, compared with 22% in cohort 2. Among those with repeat colonoscopy, the median time until first repeat colonoscopy was 38 months in cohort 1 and 64 months in cohort 2. The mean age at first repeat colonoscopy was higher in cohort 2 (70 years) compared with cohort 1 (68 years).

**Table 2.** Characteristics of persons in cohort 1 and cohort 2^a^

	Cohort 1 (21.1%)	Cohort 2 (78.9%)
Persons, n	193,745	723,880
Age at screening colonoscopy (yr)		
Mean (SD)	65.0 (7.2)	63.9 (7.1)
Median (IQR)	64 (59–70)	63 (58–69)
55–64, n (%)	99,377 (51.3)	415,755 (57.4)
65–74, n (%)	72,578 (37.5)	241,041 (33.3)
75+, n (%)	21,790 (11.3)	67,084 (9.3)
Proportion women	42.9%	58.2%
Months of follow-up Median (IQR)	64 (30–105)	69 (33–109)
Repeat colonoscopy during follow-up, n (%)	90,486 (46.7)	161,712 (22.3)
Months until first repeat colonoscopy Median (IQR)	38 (23–53)	64 (41–91)
Age at first repeat colonoscopy		
Mean (SD)	68.1 (6.9)	69.6 (6.8)
Median (IQR)	68 (62–73)	69 (64–75)
55–64, n (%)	31,809 (35.2)	42,388 (26.2)
65–74, n (%)	41,214 (45.6)	78,446 (48.5)
75+, n (%)	17,463 (19.3)	40,878 (25.3)

IQR, interquartile range; SD, standard deviation.

a Cohort 1: persons with a code for snare polypectomy (reimbursable for lesions >5 mm); cohort 2: persons without codes indicating polyps/polypectomy.

**Figure 1.**
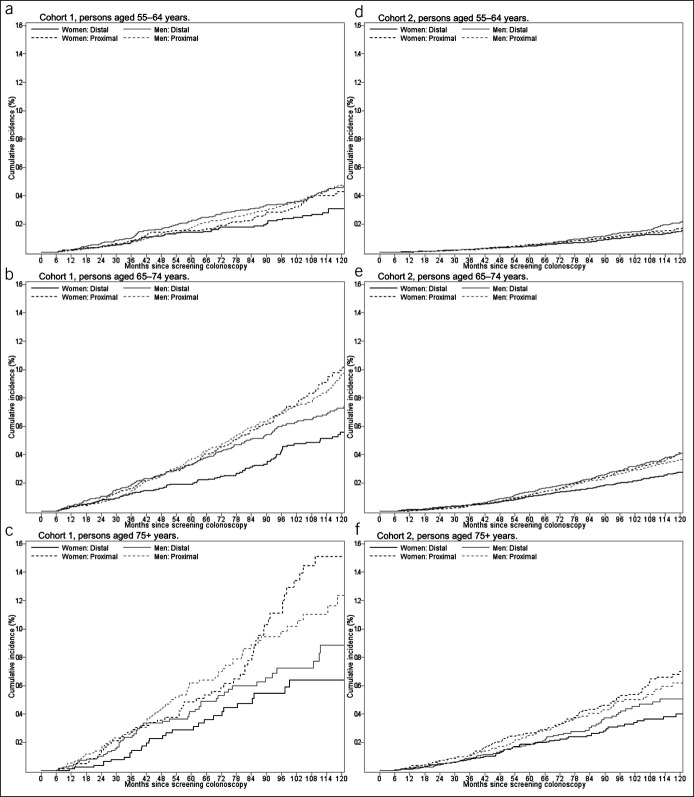
Cumulative incidence of proximal and distal colorectal cancer (CRC) according to time since screening colonoscopy for cohort 1 and cohort 2^1^ by age group and sex. ^1^Cohort 1: persons with a code for snare polypectomy (reimbursable for lesions >5 mm); cohort 2: persons without codes indicating polyps/polypectomy.

**Figure 2.**
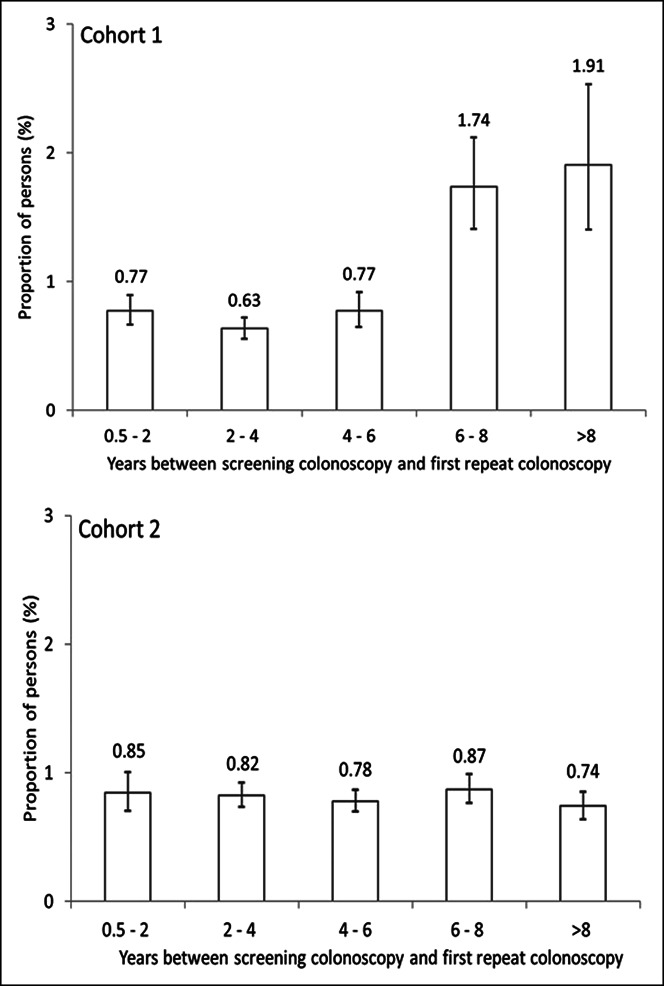
Proportion of persons with a colorectal cancer (CRC) detected at first repeat colonoscopy according to years^1^ since baseline screening colonoscopy in cohort 1 and cohort 2^2^. ^1^The time intervals did not overlap. In the category 4–6 years, for example, persons undergoing a repeat colonoscopy after 6.0 years are still included, whereas persons with a repeat colonoscopy within 6.1 years are considered in the category 6–8 years. ^2^Cohort 1: persons with a code for snare polypectomy (reimbursable for lesions >5 mm); cohort 2: persons without codes indicating polyps/polypectomy.

**Figure 3.**
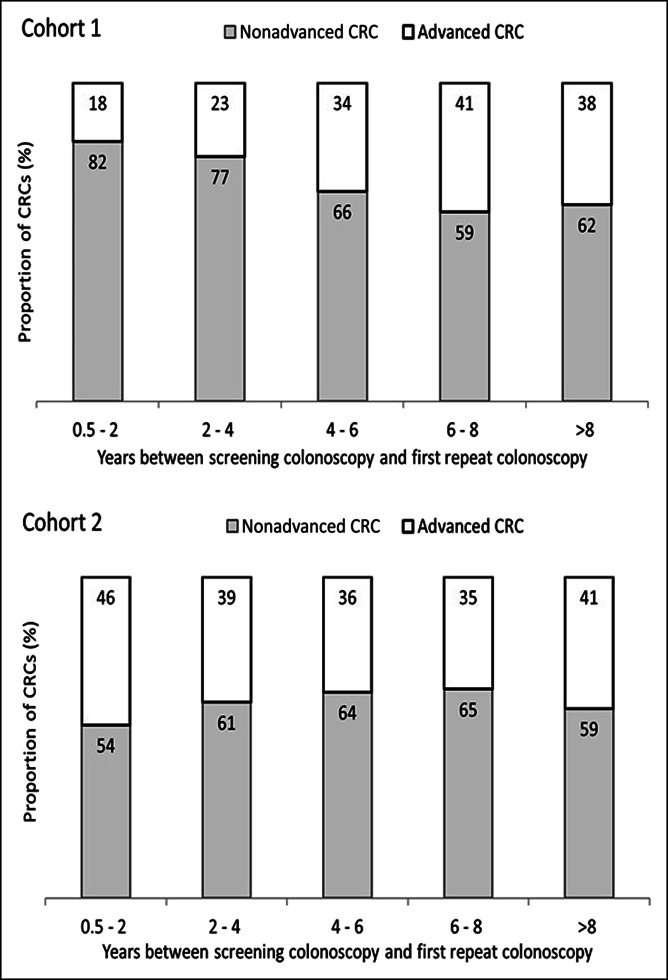
Stage distribution (advanced vs nonadvanced) of colorectal cancers (CRCs) detected at first repeat colonoscopy according to years^1^ since baseline screening colonoscopy, stratified by subcohort^2^. ^1^The time intervals did not overlap. In the category 4–6 years, for example, persons undergoing a repeat colonoscopy after 6.0 years are still included, whereas persons with a repeat colonoscopy within 6.1 years are considered in the category 6–8 years. ^2^Cohort 1: persons with a code for snare polypectomy (reimbursable for lesions >5 mm); cohort 2: persons without codes indicating polyps/polypectomy.

During follow-up, 1,481 CRCs occurred in cohort 1 and 2,400 in cohort 2. In cohort 1, 31% of these CRCs were diagnosed at an advanced stage while in cohort 2, 40% were at an advanced stage. Among women, the overall CRC incidence per 10,000 person-years in cohort 1 was 12.5 (men: 14.3) and in cohort 2, it was 5.3 (men: 5.9) (see Supplementary Digital Content 3, http://links.lww.com/CTG/A883). In both sexes and both cohorts, the proportion of proximal CRC among all CRCs occurring within 10 years increased with age at baseline (Table 3). In women of cohort 1 aged 55–64 years at baseline, the proportion of proximal CRCs among all CRCs diagnosed during follow-up was 56% (category “both/unknown” is not considered in the denominator of this proportion). In women of cohort 1 aged 65–74 years at baseline, this proportion was 63%, and in those aged 75 years or older, it was 67%. Among men of cohort 1, the proportions of proximal CRCs were 8%–9% lower compared with women in all age groups. In women of cohort 2 aged 55–64 years, 65–74 years, and 75 years or older at baseline, respectively, the proportions of proximal CRC among all CRCs diagnosed during follow-up were 53%, 58%, and 62%. In men, these proportions were 7%–11% lower. A detailed description of the cumulative incidence of proximal and distal CRCs over time—stratified by sex and age group—is presented in Figure 1a–c for cohort 1 and in Figure 1d–f for cohort 2. Sensitivity analysis subdividing distal CRCs into cancers in the distal colon vs rectal cancer showed that the proportion of rectal cancers among distal CRCs occurring during follow-up was similar across age groups (see Supplementary Digital Content 4, http://links.lww.com/CTG/A883). Figure 2 shows that the CRC detection rate at first repeat colonoscopy increased according to the time interval since screening colonoscopy in cohort 1. This increase was most pronounced after 6 years: 1.7% of those with a first repeat colonoscopy within 6–8 years had a CRC detected at first repeat colonoscopy as compared with 0.8% of those with a first repeat colonoscopy within 4–6 years. Analyses stratified by sex showed that this abrupt increase was observed both in men and women, and the increase was higher in women than in men (see Supplementary Digital Content 6, http://links.lww.com/CTG/A883). In cohort 2, there was no relevant variation in the CRC detection rate according to time since screening colonoscopy. We observed similar patterns in cohorts 1 and 2 when we stratified the analysis by proximal and distal CRCs (see Supplementary Digital Content 6, http://links.lww.com/CTG/A883). As presented in Supplementary Digital Content 7 (see Supplement, http://links.lww.com/CTG/A883), the mean age of persons in the denominator of the proportions shown in Figure 2 increased by 1–2 years per time interval in both cohorts (as expected) and the sex distribution was similar across time intervals, i.e., there was no relevant variation in the age and sex distribution across time intervals. Furthermore, the patterns shown in Figure 2 did not change if the analysis was restricted to persons aged 70 years or younger (see Supplementary Digital Content 8, http://links.lww.com/CTG/A883). Figure 3 shows the distribution of stage (advanced vs nonadvanced) of CRCs detected at first repeat colonoscopy, stratified by time since screening colonoscopy. In cohort 1, the proportion of CRCs detected at an advanced stage increased according to time since screening colonoscopy, from approximately 20% in years 1–4 to approximately 40% after more than 6 years. In cohort 2, the proportion of advanced stages ranged from 35% to 46% without a clear pattern over time.

**Table 3.** Cumulative incidence of total, proximal, and distal^a^ colorectal cancers (CRCs) at 10 years after screening colonoscopy in cohort 1 and cohort 2^b^ by age group and sex

	Cumulative incidence, n (%)				Proportion of total CRC^c^	
	Total	Proximal	Distal	Both/unknown	Proximal	Distal
Men						
Cohort 1						
55–64 yr	286 (1.1%)	120 (0.5%)	130 (0.5%)	36 (12.6%)	48.0%	52.0%
65–74 yr	442 (1.9%)	218 (1.0%)	183 (0.7%)	41 (9.3%)	54.4%	45.6%
75+ yr	138 (2.4%)	71 (1.2%)	52 (0.9%)	15 (10.9%)	57.7%	42.3%
Cohort 2						
55–64 yr	330 (0.4%)	121 (0.2%)	155 (0.2%)	54 (16.4%)	43.8%	56.2%
65–74 yr	510 (0.9%)	212 (0.4%)	236 (0.4%)	62 (12.2%)	47.3%	52.7%
75+ yr	163 (1.2%)	84 (0.6%)	68 (0.5%)	11 (6.7%)	55.3%	44.7%
Women						
Cohort 1						
55–64 yr	175 (0.9%)	82 (0.4%)	64 (0.3%)	29 (16.6%)	56.2%	43.8%
65–74 yr	277 (1.8%)	153 (1.0%)	89 (0.6%)	35 (12.6%)	63.2%	36.8%
75+ yr	100 (2.4%)	58 (1.5%)	29 (0.6%)	13 (13.0%)	66.7%	33.3%
Cohort 2						
55–64 yr	441 (0.4%)	182 (0.2%)	160 (0.1%)	99 (22.4%)	53.2%	46.8%
65–74 yr	584 (0.8%)	289 (0.4%)	209 (0.3%)	86 (14.7%)	58.0%	42.0%
75+ yr	198 (1.2%)	115 (0.7%)	70 (0.4%)	13 (6.6%)	62.2%	37.8%

a Numbers of proximal + distal CRC do not add up to total CRC because of persons in the group of “both/unknown” location.

b Cohort 1: persons with a code for snare polypectomy (reimbursable for lesions >5 mm); cohort 2: persons without codes indicating polyps/polypectomy.

c Excluding those in the category “both/unknown” in the denominator.

## DISCUSSION

Our study including 1 million persons undergoing a screening colonoscopy followed for a period of up to 10 years provides, for the first time, detailed insights into the occurrence of distal and proximal CRCs among men and women of different age groups after screening colonoscopy. Although there was an overall trend toward higher incidences for proximal as compared with distal CRC during follow-up in men and women, we observed distinct differences by age and sex. In women, the proportion of postcolonoscopy CRCs located in the proximal colon was more than 50% already in those aged 55–64 years at baseline and was 60% or higher in older women. In men, the proportion of postcolonoscopy CRCs located in the proximal colon was 7%–11% lower across all age groups. Our analysis focusing on first repeat colonoscopy according to time since baseline screening colonoscopy showed a sharp rise in the CRC detection rate (proximal and distal) among persons with snare polypectomy if repeat colonoscopy was performed after more than 6 years: Compared with persons undergoing a repeat colonoscopy after 4–6 years, it was 2 times higher in men and even 3 times higher in women, and the CRC detection rate was even higher than that at baseline screening colonoscopy. In addition, the proportion of advanced CRCs increased according to the time interval since baseline in persons with polypectomy. In persons without polypectomy at baseline, the pattern was different. There was no increase in the CRC detection rate over time, and the proportion of advanced CRC was constant, but at a remarkably high level (∼40%).

While several other studies investigated the cumulative CRC incidence by time since baseline colonoscopy (2,3,20,21), there is a lack of knowledge regarding the CRC detection rate depending on the timing of the first repeat colonoscopy. Only 1 recently published study from the United Kingdom reported on CRC detection rates at first surveillance colonoscopy according to the time interval since baseline colonoscopy, but it was limited to 30,000 persons with adenomas at baseline. Owing to the small sample size (322 CRCs detected at first surveillance colonoscopy overall), careful interpretation is needed, but it was still striking that the CRC detection rate was more than 3 times higher in those with an interval of >6 years between baseline and first surveillance as compared with those with an earlier surveillance (22).

There is an ongoing discussion on optimal surveillance intervals for persons with low-risk adenomas and the definition of low-risk. Current recommendations for low-risk vary between guidelines. For example, the US Multi-Society Task Force on Colorectal Cancer recommends an interval of 7–10 years (23) and the European Society of Gastrointestinal Endoscopy recommends 10 years (24). Of note, both guidelines stress that these recommended intervals apply only to baseline colonoscopies with a high quality, which is often not the case in the real-world setting (25). The German guidelines used to recommend 5 years (16), which has been changed to 5–10 years in 2017 (26). Our study is not suited to provide evidence on how to optimize surveillance intervals, particularly because we did not have information on the number, histology, and exact size of adenomas. However, it is quite certain that almost all persons with snare polypectomy at baseline in our study had a recommended surveillance interval of 3 or 5 years according to guideline recommendations applicable during the study period (16). Persons with snare polypectomy undergoing repeat colonoscopy after more than 6 years were thus not treated according to these recommendations. CRCs occurring in this group might have been avoided if the recommendations had been followed. In a previous analysis, we were able to show that approximately 35% of persons with a snare polypectomy at screening colonoscopy in Germany did not undergo a repeat colonoscopy within 6 years (15). Implementing a reminder system for these persons to undergo surveillance colonoscopy as recommended by guidelines might be a promising strategy. It would be worthwhile to explore whether this could improve the effectiveness of the CRC screening program in Germany. It would also be a pragmatic option given that all information needed for such an intervention—i.e., whether and when screening colonoscopy with snare polypectomy was performed and whether there was a repeat colonoscopy—is available at the health insurance providers which are also in charge of sending out the primary invitation letters for CRC screening in Germany.

Regarding persons without polypectomy at screening colonoscopy, our study demonstrates that CRC detection rates did not increase with longer time intervals since screening colonoscopy and the proportion of advanced CRC remained stable. These results support surveillance guidelines recommending a repeat colonoscopy not earlier than 10 years after a negative colonoscopy (27). In a previous analysis, we were able to show that these recommendations are not being followed by a significant proportion of screening participants: 43% of persons without polypectomy at baseline had a repeat colonoscopy within less than 10 years (15). According to our results, this substantial overuse is not expected to have a beneficial effect on CRC incidence or mortality.

In our study, there were also CRCs occurring within a shorter time period after colonoscopy. We did not have the required data to analyze the potential explanations for these postcolonoscopy CRCs as previously described (18). The results of previous studies using such an approach suggest that CRCs occurring within 5 years could mostly be avoided. For example, the study by le Clercq et al. (28) examining the likely etiology of 5,107 patients with CRC diagnosed within 5 years (on average after 26 months) after an index colonoscopy estimated that 67% of the postcolonoscopy CRCs were attributed to missed lesions or incomplete resection, 20% to inadequate examination/surveillance, and only 14% were assumed to have newly developed.

Our study also provides, for the first time, detailed information on the long-term incidence of distal vs proximal CRC after screening colonoscopy stratified by age and sex. While the distribution across all subgroups (47% proximal vs 39% distal CRCs) is in line with other studies showing a predominance of proximal CRCs after an index colonoscopy (2–6,8,9), our subgroup analyses showed that there are marked differences by age and sex. Particularly in men, there was a clear predominance of proximal CRC throughout follow-up only in those already at an older age at baseline screening colonoscopy. The patterns by age and sex were similar when we focused on CRCs detected at the first repeat colonoscopy rather than on the cumulative CRC incidence (see Supplementary Digital Content 5, http://links.lww.com/CTG/A883), suggesting that adenoma removal at surveillance colonoscopy conducted during follow-up did not markedly change the overall distribution. Interestingly, the distinct patterns by age and sex regarding the occurrence of distal vs proximal CRC observed in our study among persons with prior screening colonoscopy agree well with the occurrence of distal and proximal CRCs in men and women without prior endoscopy. For example, a pooled analysis of randomized controlled trials on the effectiveness of flexible sigmoidoscopy showed a general shift toward more proximal CRCs at older age in the usual care group but among women, this shift was at a younger age than among men (≥60 vs ≥ 70 years) (11). This underlines the hypothesis that there are certain pathways to CRC becoming particularly relevant at an older age (earlier in women than in men) and affecting mainly the proximal colon. Further research on the molecular basis of these patterns including the role of the CpG methylator phenotype and microsatellite instability may be worthwhile also in view of the development of potential biomarkers that may predict postcolonoscopy CRC.

Of note, whether or to which extent CRCs in the proximal and distal colon are preventable through colonoscopy cannot be assessed based on our study, which was merely descriptive. It would require an appropriately designed study that compares the effectiveness of colonoscopy in reducing distal vs proximal CRC incidence. A recently published study addressing this research question based on an emulated target trial did not observe relevant differences in the effectiveness by location (29). It is thus not appropriate to conclude that colonoscopy is less effective in reducing the incidence of proximal CRC, simply from descriptive analyses showing a predominance of proximal CRC in persons with prior endoscopy.

The overall 10-year cumulative CRC incidence observed in our study (1.5% among persons with snare polypectomy and 0.6% among persons without polypectomy at baseline) seem somewhat high compared with that in other studies (3,20). The study by Lee et al. (20) including 64,422 persons with colonoscopy reported a 10-year cumulative CRC incidence of 1.2% in the high-risk adenoma group and 0.4% in the low-risk adenoma group. The study by He et al. (3) including 122,899 persons with flexible screening sigmoidoscopy or colonoscopy reported a 10-year cumulative incidence of 1.7% in persons with advanced adenoma and 0.3% in persons with nonadvanced adenoma. Most likely, these differences can be explained by the more restrictive exclusion criteria and the lower mean age in these studies compared with our study. As presented in Supplementary Digital Content 1 (see Supplement, http://links.lww.com/CTG/A883), CRC incidence is not overestimated in GePaRD.

When interpreting our results, it should be noted that our study and the database we used have strengths and limitations. As we used claims data, the analyses are free of recall and volunteer bias and the large sample size facilitated analyses stratified by tumor site, age, and sex. Furthermore, the database includes precise and complete information on utilization of repeat colonoscopy, which—in combination with the large sample size—facilitated analyses that have never been done before. Our database does not include information on histology of polyps detected at colonoscopy, but we could at least distinguish between persons with snare polypectomy (reimbursable for polyps >5 mm in size) and persons without polypectomy, showing clearly different rates of CRC incidence during follow-up. Furthermore, claims data do not contain direct information on the quality of colonoscopy or information on completeness of polypectomy. However, according to the German screening colonoscopy registry, the cecal intubation rate is >90% and ∼92% of polypectomies are complete (30). In addition, the information on CRC diagnoses may not be perfect in claims data. However, in more than 90% of the patients with CRC diagnosis during follow-up in our study, the disease was coded in the inpatient setting, i.e., a setting providing very valid information and specific *ICD-10* codes, which are needed to classify tumor location. For the remaining patients, we used strict criteria to minimize misclassification. The high agreement between GePaRD and cancer registry data regarding CRC incidence, the proportion of advanced stages, and the distribution by location supports the validity of the case definitions we used (see Supplementary Digital Content 1, http://links.lww.com/CTG/A883). There was a small proportion of CRCs with missing information on location, but there is no reason to assume that these missing values concern one location more than the other, i.e., a distortion of the distribution by location due to these missing values seems unlikely.

In conclusion, our study among persons with screening colonoscopy at baseline followed over 10 years showed a steadily increasing predominance of proximal CRC during follow-up, and this shift showed distinct patterns by age and sex. Among persons with snare polypectomy at baseline, the CRC detection rate at first repeat colonoscopy abruptly increased when the examination was performed more than 6 years after baseline. Further studies are needed that explore whether these CRCs could be avoided by interventions aiming to avoid delays in the uptake of surveillance colonoscopy as recommended according to baseline findings.

## CONFLICTS OF INTEREST

**Guarantor of the article:** Ulrike Haug, PhD.

**Specific author contributions:** S.S. and U.H.: conceptualized the study and developed the data analysis plan. S.S. and M.H.: contributed to data analysis. S.S.: drafted the first version of the manuscript. All authors contributed to interpretation of the results and critically revised the manuscript draft. All authors approved the final version of the manuscript.

**Financial support:** None to report.

**Potential competing interests:** None to report.

**Study Highlights**

**WHAT IS KNOWN**

✓ Colorectal cancers (CRCs) also occur after colonoscopy.
✓ Knowledge on the epidemiology of postcolonoscopy CRCs by location and on CRC detection rates at first repeat colonoscopy is limited.

**WHAT IS NEW HERE**

✓ We observed a steadily increasing predominance of proximal CRC after screening colonoscopy, especially in older age groups and women.
✓ We found a sharp rise in the CRC detection rate if the first surveillance colonoscopy was performed more than 6 years after polypectomy.
✓ The findings provide important insights into the epidemiology of postcolonoscopy colorectal cancer.
✓ They highlight the risk of delays in surveillance and provide a concrete, scientific basis for planning interventions to avoid such delays.

## References

[1] Brenner H Stock C Hoffmeister M. Effect of screening sigmoidoscopy and screening colonoscopy on colorectal cancer incidence and mortality: Systematic review and meta-analysis of randomised controlled trials and observational studies. BMJ 2014;348(1):g2467. 24922745 10.1136/bmj.g2467 PMC3980789

[2] Click B Pinsky PF Hickey T Association of colonoscopy adenoma findings with long-term colorectal cancer incidence. JAMA 2018;319(19):2021–31. 29800214 10.1001/jama.2018.5809 PMC6583246

[3] He X Hang D Wu K Long-term risk of colorectal cancer after removal of conventional adenomas and serrated polyps. Gastroenterology 2020;158(4):852–61.e4. 31302144 10.1053/j.gastro.2019.06.039 PMC6954345

[4] Nishihara R Wu K Lochhead P Long-term colorectal-cancer incidence and mortality after lower endoscopy. N Engl J Med 2013;369(12):1095–105. 24047059 10.1056/NEJMoa1301969 PMC3840160

[5] Lakoff J Paszat LF Saskin R Risk of developing proximal versus distal colorectal cancer after a negative colonoscopy: A population-based study. Clin Gastroenterol Hepatol 2008;6(10):1117–21. 18691942 10.1016/j.cgh.2008.05.016

[6] Lee JK Jensen CD Levin TR Long-term risk of colorectal cancer and related deaths after a colonoscopy with normal findings. JAMA Intern Med 2019;179(2):153–60. 30556824 10.1001/jamainternmed.2018.5565 PMC6439662

[7] Pilonis ND Bugajski M Wieszczy P Long-term colorectal cancer incidence and mortality after a single negative screening colonoscopy. Ann Intern Med 2020;173(2):81–91. 32449884 10.7326/M19-2477

[8] Samadder JN Pappas L Boucherr KM Long-term colorectal cancer incidence after negative colonoscopy in the state of Utah: The effect of family history. Am J Gastroenterol 2017;112(9):1439–47. 28695908 10.1038/ajg.2017.193

[9] Singh H Nugent Z Mahmud SM Predictors of colorectal cancer after negative colonoscopy: A population-based study. Am J Gastroenterol 2010;105(3):663–73. 19904239 10.1038/ajg.2009.650

[10] Song M Emilsson L Bozorg SR Risk of colorectal cancer incidence and mortality after polypectomy: A Swedish record-linkage study. Lancet Gastroenterol Hepatol 2020;5(6):537–47. 32192628 10.1016/S2468-1253(20)30009-1 PMC7234902

[11] Holme Ø Schoen RE Senore C Effectiveness of flexible sigmoidoscopy screening in men and women and different age groups: Pooled analysis of randomised trials. BMJ 2017;356:i6673. 28087510 10.1136/bmj.i6673 PMC5234564

[12] Overbeek JA Kuiper JG van der Heijden AAWA Sex- and site-specific differences in colorectal cancer risk among people with type 2 diabetes. Int J Colorectal Dis 2019;34:269–76. 30421309 10.1007/s00384-018-3191-7 PMC6331739

[13] Iida Y Kawai K Tsuno NH Proximal shift of colorectal cancer along with aging. Clin Colorectal Cancer 2014;13(4):213–8. 25245544 10.1016/j.clcc.2014.06.005

[14] McCashland TM Brand R Lyden E Gender differences in colorectal polyps and tumors. Am J Gastroenterol 2001;96(3):882–6. 11280569 10.1111/j.1572-0241.2001.3638_a.x

[15] Schwarz S Schäfer W Horenkamp-Sonntag D Follow-up of 3 million persons undergoing colonoscopy in Germany: Utilization of repeat colonoscopies and polypectomies within 10 years. Clin Transl Gastroenterol 2021;12(1):e00279. 10.14309/ctg.0000000000000279 PMC8345921 33464730

[16] Pox C Aretz S Bischoff SC S3-guideline colorectal cancer version 1.0. Z Gastroenterol 2013;51(8):753–854. 23955142 10.1055/s-0033-1350264

[17] Oppelt KA Luttmann S Kraywinkel K Incidence of advanced colorectal cancer in Germany: Comparing claims data and cancer registry data. BMC Med Res Methodol 2019;19:142–9. 31286896 10.1186/s12874-019-0784-y PMC6615087

[18] Rutter MD Beintaris I Valori R World Endoscopy Organization consensus statements on post-colonoscopy and post-imaging colorectal cancer. Gastroenterology 2018;155(3):909–25.e3. 29958856 10.1053/j.gastro.2018.05.038

[19] Daly LE. Confidence limits made easy: Interval estimation using a substitution method. Am J Epidemiol 1998;147(8):783–90. 9554420 10.1093/oxfordjournals.aje.a009523

[20] Lee JK Jensen CD Levin TR Long-term risk of colorectal cancer and related death after adenoma removal in a large, community-based population. Gastroenterology 2020;158(4):884–94.e5. 31589872 10.1053/j.gastro.2019.09.039 PMC7083250

[21] Wieszczy P Kaminski MF Franczyk R Colorectal cancer incidence and mortality after removal of adenomas during screening colonoscopies. Gastroenterology 2020;158(4):875–83.e5. 31563625 10.1053/j.gastro.2019.09.011

[22] Cross AJ Robbins EC Pack K Colonoscopy surveillance following adenoma removal to reduce the risk of colorectal cancer: A retrospective cohort study. Health Technol Assess 2022;26:1–156. 10.3310/OLUE3796 PMC9376986 35635015

[23] Gupta S Lieberman D Anderson JC Recommendations for follow-up after colonoscopy and polypectomy: A consensus update by the US Multi-Society Task Force on Colorectal Cancer. Gastrointest Endosc 2020;91(3):463–85.e5. 32044106 10.1016/j.gie.2020.01.014 PMC7389642

[24] Hassan C Antonelli G Dumonceau J-M Post-polypectomy colonoscopy surveillance: European Society of Gastrointestinal Endoscopy (ESGE) guideline–update 2020. Endoscopy 2020;52(08):687–700. 32572858 10.1055/a-1185-3109

[25] Wieszczy P Regula J Kaminski MF. Adenoma detection rate and risk of colorectal cancer. Best Pract Res Clin Gastroentero 2017;31(4):441–6. 10.1016/j.bpg.2017.07.002 28842054

[26] Schmiegel W Buchberger B Follmann M S3-guideline colorectal cancer version 2.0. Z Gastroenterol 2017;55(12):1344–498. 29212104 10.1055/s-0043-121106

[27] German Guideline Program in Oncology (German Cancer Society GCA, AWMF). S3-Guideline Colorectal Cancer, Long Version 2.1, AWMF Registrationnumber: 021-007OL, 2019.

[28] le Clercq CMC Bouwens MWE Rondagh EJA Postcolonoscopy colorectal cancers are preventable: A population-based study. Gut 2014;63(6):957–63. 23744612 10.1136/gutjnl-2013-304880

[29] Braitmaier M Schwarz S Kollhorst B Screening colonoscopy similarly prevented distal and proximal colorectal cancer; A prospective study among 55-69-year-olds. J Clin Epidemiol 2022;149:118–26. 35680106 10.1016/j.jclinepi.2022.05.024

[30] Kretschmann J El Mahi C Lichtner F Früherkennungskoloskopie. Jahresbericht 2019. Zentralinstitut für die kassenärztliche Versorgung in Deutschland: Berlin, Germany, 2021.

